# iWorkHealth: An instrument to identify workplace psychosocial risk factors for a multi-ethnic Asian working population

**DOI:** 10.1371/journal.pone.0220566

**Published:** 2019-08-07

**Authors:** Edimansyah Abdin, Mythily Subramaniam, Angelina Chan, Jo-Ann Chen, Chee Leong Chong, Cheryl Wang, Michelle Lee, Siok Lin Gan

**Affiliations:** 1 Institute of Mental Health, Singapore, Singapore; 2 Changi General Hospital, Singapore, Singapore; 3 Ministry of Manpower, Singapore, Singapore; 4 Clanworks, Singapore, Singapore; 5 Work Safety and Health Council, Singapore, Singapore; 6 Health Promotion Board, Singapore, Singapore; Mohammed bin Rashid University of Medicine and Health Sciences, UNITED ARAB EMIRATES

## Abstract

**Objective:**

The current study aimed to develop iWorkHealth, a valid and reliable self-administered instrument which identifies workplace psychosocial risk factors in Singapore.

**Methods:**

The survey was conducted among 2718 employees who were primarily salaried workers and working in five companies from the healthcare, banking and finance, and legal sectors in Singapore. Factor extraction and item reduction were conducted using exploratory factor analysis (EFA) and Mokken scale analysis (MSA). Construct validity, internal consistency and convergent validity of the final scale were confirmed using confirmatory factor analysis (CFA), Cronbach's alpha and Pearson correlation coefficients, respectively. Multiple Indicators Multiple Causes model was used to detect Differential Item Functioning (DIF).

**Results:**

EFA and MSA identified a five-factor solution (job demand, job control, employee and management engagement, supervisor support and colleague support) for the 27 items iWorkHealth instrument. CFA demonstrated that the five-factor model fitted the data with high internal consistency (Cronbach's alpha ranged from 0.79 to 0.92). The convergent validity was shown through significant association with existing scales—high job demand was significantly associated with high burnout and depression, while high job control, employee and management engagement, supervisor support and coworker support were significantly associated with low burnout and depression. Ten items were detected with significant DIF, but impact was minimal on the associations between socio-demographics factors and iWorkHealth subscales.

**Conclusions:**

The findings provided evidence that the iWorkHealth instrument which comprises 27 items in five domains of psychosocial risk at the workplace is a reliable and valid instrument that could be used to measure and compare the level of psychosocial risk factors across companies and industries in Singapore.

## Introduction

The increasing presence of workplace psychosocial risk factors such as poor organizational climate, introduction of new technologies, renewed business models, social relationship and leadership, work-family conflict, high work pressure, and job insecurity has resulted in significant negative impact on employees' psychological wellbeing, physical health and safety, as well as organizational performance-related outcomes such as absenteeism, job dissatisfaction and loss of productivity [1, 2]. The International Labor Organization [3] defines psychosocial risk at work as interactions between the individual and a range of workplace factors including job design, management, and the organizational environment that have the potential to have a hazardous influence on employee’s health. A hazard occurs when one or more of these factors have a detrimental effect on a worker’s wellbeing, resulting in poor health outcomes such as exhaustion, anxiety or depression [4]. To manage any of these psychosocial risk factors at work, it is crucial to first determine their existence and prevalence followed by identifying the specific groups at risk [5].

Over the past decades, various instruments have been developed that measure psychosocial risk factors at the workplace including the Job Content Questionnaire [6], the Effort-Reward Imbalance Questionnaire [7] and the Copenhagen Psychosocial Questionnaire [8]. In other countries, especially those in Asia, there are cultural differences and concepts of psychosocial risk factors may be perceived differently and even uniquely depending on the population [9]. Hence, it is important to adopt some of these key instruments and use their items to specifically develop an instrument that is relevant for use in a culturally diverse population that recognizes how within that population, the various cultural subgroups may think, feel and act differently on various issues at work [10].

This research focuses on Singapore which is an island city-state in Southeast Asia with a multi-ethnic Asian population of approximately 5.64 million people in 2018. The population comprises Chinese (74.3%), Malays (13.4%), Indians (9.0%), and other ethnic groups (3.2%) [11]. It has a predominantly working population where 65.1% of residents aged 15 and over were employed in 2018 [12]. Hence, it is not surprising that work-related stress, psychological, physical and behavioural problems such as depression, high blood pressure, burnout, absenteeism, turnover intention etc., are being recognised as an emerging area of concern in this multi-ethnic Asian working population. However, a valid and reliable instrument to assess psychosocial risk factors in this working population, which is applicable across companies and industries, is currently lacking. Moreover, few studies have considered measurement bias such as differential item functioning (DIF) when developing a psychosocial risk factor instrument [13]. Hence, the current study aimed to develop a self-administered instrument that embraces the multi-faceted workplace psychosocial risk in Singapore.

## Material and methods

### Study design and participants

**Fig 1:**
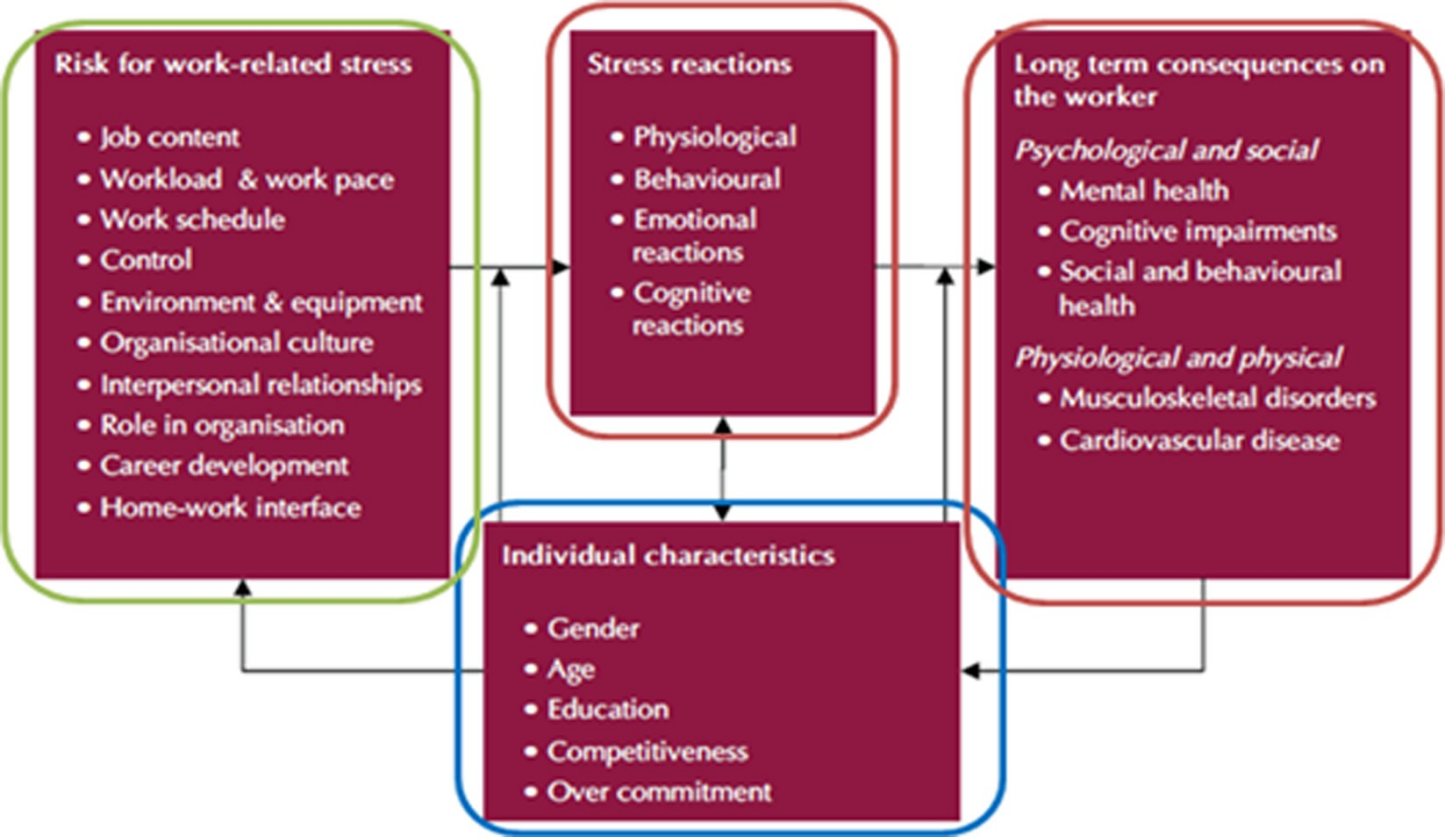
Model of psychosocial risk factors and its probable outcomes.

This multiphase study involved the development and validation of the instrument in a phased manner. In the development phase, the research team conducted an extensive literature review to identify the plausible factors that influence an employee’s psychosocial risk (Fig 1) and derived a model of psychosocial risk factors and outcomes. Instruments relevant to measuring these psychosocial risk factors were then identified and all the researchers proceeded to select the appropriate scales and items through collaborative discussions for the draft version of the instrument.

Next, the draft instrument was field-tested in focus group discussions (FGDs) where a total of 29 employees belonging to different sectors and educational qualifications participated. The participants were also encouraged to identify factors that they felt contributed to psychosocial risk but had not been included. They were also asked to identify those that they felt were not relevant. In addition, participants also provided input on the phrasing and sentence construction of the questionnaire and the response scales.

In the validation phase, a cross-sectional study was conducted among employees aged 18 years and above who were working in Singapore. Research data were collected using a questionnaire survey from five companies operating in the healthcare, banking and finance and legal sectors. The most common types of jobs among participants were nurses, allied health professionals and lawyers. A total of 2718 employees from the three sectors participated with response rates ranging from 70% (banking industry) to 84% (healthcare industry). However, only 2713 were included in the current analyses after excluding five respondents aged below 20 years. The sample comprised twice as many females as males (69.1% vs 30.9%). The majority were Chinese (65.2%) followed by Malays (15.8%), Indians (11%), and other ethnic groups (8%). Education ranged from Primary school (≤ 6 years of education) to Master/Doctorate, with the majority having a Bachelor degree (43.4%), followed by Secondary education (16.1%), Diploma (15%) and Master/Doctorate degree (14.4%) (Table 1). The ethical approval for the study was obtained from the SingHealth Centralised Institutional Review Board in Singapore. Waiver for informed consent was approved. Implicit consent was obtained from all participants using a participant information sheet which was included with the data collection form. The participant information sheet provided all the study details on the voluntary nature and risk benefits of the study. Contact numbers of Principal Investigator and Co-Investigators were also provided in case the participants had any other related queries. Diligent care was exercised to ensure that no identifiers were collected from the participants.

**Table 1:** Sociodemographic characteristics of the sample.

Gender	n	%
Men	784	30.9
Women	1753	69.1
Age group		
20 to 29 years	597	23.5
30 to 39 years	881	34.6
40 to 49 years	565	22.2
50 to 59 years	353	13.9
60 to 69 years	138	5.4
70 years and above	10	0.4
Ethnicity		
Chinese	1650	65.2
Malay	400	15.8
Indian	278	11.0
Others	203	8.0
Marital status		
Single	923	36.4
Married	1499	59.1
Divorced or Separated	89	3.5
Widowed	26	1.0
Highest education level		
Primary Education / PSLE & Below	22	0.9
Secondary education / ‘N’ or ‘O’ Levels	408	16.1
Post-Secondary education / ITE NITEC / Higher NITEC / ‘A’ Levels	201	8.0
Diploma	379	15.0
Bachelor’s Degree	1099	43.4
Postgraduate Diploma / Certificate Qualification (excluding Master’s / Doctorate)	61	2.4
Master’s / Doctorate	365	14.4
Sector		
Healthcare	1698	62.59
Legal	819	30.1
Banking	196	7.2

PSLE: Primary School Leaving Examination; ‘N’: Singapore-Cambridge General Certificate of Education Normal Level; ‘O’: Singapore-Cambridge General Certificate of Education Ordinary Level; ‘A’: Singapore-Cambridge General Certificate of Education Advanced Level; ITE: Institute of Technical Education; NITEC: National ITE Certificate

### Instruments

Demographic data, psychosocial outcomes, and items to assess psychosocial risk factors were included as part of the questionnaire. Demographic data included age, gender, ethnicity, marital status and highest education level attained.

Psychosocial outcomes were measured using the following two scales:

- Copenhagen Burnout Inventory (CBI) [14]. This has three sub-dimensions, namely personal burnout, work-related burnout and client-related burnout. The personal burnout subscale is generic, for all participants to be able to respond. The work-related burnout subscale assumes that respondents are engaged in some kind of paid work and the client-related burnout subscale includes questions with the term client, which can be replaced by terms such as colleague, customer, or patient [8].
- Patient Health Questionnaire-2 (PHQ-2) [15]. This comprises the first two items of PHQ-9 and looks at the frequency of depressed mood and anhedonia over the past two weeks. Score ranges from 0–6 with higher scores indicative of more severe depressive symptoms.

45 items of psychosocial risk factors were identified from the literature search and FGDs in the development phase, with participants responding using a five-point Likert scale, where ‘1’ represented ‘strongly disagree’ and ‘5’ corresponded to ‘strongly agree’.

### Statistical analysis

A series of steps were taken to develop and validate the scale. To obtain the underlying factors, a spilt-half test was first done where the 2713 participants were randomly divided into two groups. Exploratory factor analysis (EFA) was used on the first subsample (n = 1356) to extract the factors. The Mokken scale analysis (MSA) was used to examine item performance and to carry out the final item reduction. The derived factors were then applied to the second subsample (n = 1357) and validated using confirmatory factor analysis (CFA). EFA examined the polychoric correlations with weighted least squares with the mean- and variance-adjusted chi-square (WLSMV) estimator. An oblique geomin rotation was then applied to obtain a more discriminating factor structure. Several criteria were used to determine the number of factors in EFA such as eigenvalues (values >1.0), visual sighting of scree plot, identifying pattern of loadings on each factor (i.e. loading > 0.4 or cross-loading items), and robustness of interpretability for each solution. The psychometric properties of each scale and its associated items were further assessed using MSA. Generally, the MSA assessed four fundamental assumptions including unidimensionality, local independence, monotonicity and non-intersection. A set of items was assumed to form a good scale if the H*i* coefficient (Loevinger’s scalability coefficient for the item) for each pair of items was greater than or equal to 0.3. The scale was classified based on H value (Loevinger’s scalability coefficient for the scale) which classified H value of 0.3 to <0.4 as indicating a weak scale, 0.4 to <0.5 a medium scale, and 0.5 to 1.0 a strong scale [16]. After determining the factor solution and eliminating items with low factor loadings and scalability index from both EFA and MSA, the construct validity of the final model was confirmed using CFA’s fit indices that included comparative fit index (CFI) >0.95, Tucker-Lewis index (TLI) >0.90, and root mean square error of approximation (RMSEA). The RMSEA value at 0.05 and below and 0.08 and below indicated good and moderate fit [17, 18]. Internal consistency of each subscale was evaluated using Cronbach’s alpha coefficient, where the acceptable level was set at 0.7 [19]. The convergent validity in overall sample and by age, gender, ethnicity and education status was examined with Pearson correlations between the psychosocial risk factors instrument and the external instrument which measured burnout and depression. In order to ensure that the items were not biased due to differences in age groups (young: 20 to 39 years, middle: 40 to 59 years, older: 60 years and above), gender (Male and Female), ethnicity (Chinese, Malay, Indian and Others), marital status (Single, Married, Divorced/Separated), education level (Secondary and below, Vocational/ITE, Diploma, Tertiary including Bachelor, Postgraduate Diploma, Master and Doctorate Degree) and sector (Healthcare, Legal and Banking); DIF tests were incorporated in the analyses. According to Zumboo [20] the instrument may exhibit DIF when the respondents of different groups endorse an item unequally given the same latent trait that the item intends to measure. Previous studies have suggested that if an instrument exhibits DIF it might lead to different results [14], and therefore, statistical adjustments such as a Multiple Indicators Multiple Causes (MIMIC) modeling should be used to address DIF and reduce its impact on group comparisons [21, 22]. Hence, MIMIC modelling was implemented to explore associations between socio-demographic factors and iWorkHealth subscales and address significant DIF in the regression model. Statistical significance was set at p value < 0.05.

## Results

### Factor extraction and item reduction

The factors identified through the literature review and FGDs included workload, job demand, job control, job meaning, job reward, organizational culture, colleague support, and supervisor support. A series of EFAs were conducted to determine the dimensionality of the instrument. The plot of eigenvalues for the initial 45-item indicated that five, six and seven factor solution were plausible. Upon examination of each of the rotated solutions including pattern of factor loadings i.e. cross-loading and loadings > 0.40, 6 items were removed and the six-factor solution with 39-item was found to be optimal. Item performance and item reductions were further tested using MSA. 12 items with poor item fit (H*i* less than 0.3) were deleted. The range of item scalability index value for the remaining 27 items ranged from 0.40 to 0.78. Re-examination of factor structure using 27 items concluded that the five-factor solution was optimal with higher factor loadings within each factor. Based on the content of the remaining items in each factor, the five factors were re-named: job demand, job control, employee and management engagement, supervisor support and colleague support. The H value for their subscales ranged from 0.462 (job demand) to 0.756 (colleague support) indicating medium to strong subscales according to Loevinger’s scalability classification criteria (Table 2).

**Table 2:** Factor loadings and item (H*i*) scalability of five factors of the model.

	Job demand	Job control	Supervisor support	Employee and management engagement	Colleague support	Item scalability
						(H*i*)
1. I feel that my workload is too heavy.	0.86	0.02	-0.02	0.07	-0.07	0.61
2. I have so much work to do that I am unable to do a good job.	0.79	-0.23	0.05	0.07	0.01	0.55
3. I still feel tired from the previous work day / shift even as I start the next one.	0.67	-0.18	0.04	-0.11	0.06	0.52
4. My work is emotionally demanding.	0.59	0.25	-0.04	-0.10	0.01	0.47
5. In my work, I experience contradictory demands.	0.57	0.14	-0.11	-0.12	-0.02	0.48
6. I know exactly what is expected of me at work.	0.00	0.50	0.10	0.03	0.05	0.40
7. I can use my skills and expertise in my job.	0.09	0.59	0.04	-0.03	0.12	0.44
8. I have enough information to get my job done.	-0.13	0.55	0.06	-0.04	0.14	0.42
9. I receive enough help and equipment to get my job done.	-0.19	0.45	0.12	0.07	0.20	0.42
10. My work is meaningful.	0.00	0.88	0.01	0.01	0.01	0.51
11. My work is important.	0.06	0.92	-0.05	0.06	-0.04	0.55
12. I feel motivated and involved in my work.	-0.03	0.65	0.08	0.21	0.00	0.51
13. I receive the respect and prestige I deserve at work.	0.04	0.04	0.16	0.58	0.14	0.54
14. I am satisfied with the amount of pay and benefits I receive.	-0.01	-0.02	-0.09	0.73	-0.01	0.47
15. I find the opportunities for promotion within the company are good.	0.13	-0.04	0.06	0.76	0.00	0.55
16. I feel that rewards for my effort are given in a fair way.	-0.04	-0.03	0.13	0.71	0.08	0.59
17. I feel this company treats its employees well.	-0.06	0.10	0.01	0.74	0.05	0.60
18. I think this company considers employee welfare much more important than operations / sales and profits.	-0.04	0.05	-0.07	0.75	-0.02	0.51
19. My company manages changes in policies / structures / processes well.	-0.01	0.12	0.00	0.59	-0.05	0.47
20. I receive support and guidance from my immediate supervisor.	-0.02	0.11	0.85	-0.02	0.00	0.76
21. My immediate supervisor is concerned about the welfare of his or her staff.	0.00	0.02	0.96	0.02	-0.06	0.77
22. My immediate supervisor is successful in getting people to work together.	-0.02	0.01	0.87	0.09	-0.03	0.76
23. I am treated with respect by my immediate supervisor.	0.00	-0.01	0.84	-0.04	0.16	0.76
24. My immediate supervisor talks with me about how well I carry out my work.	0.01	0.02	0.73	0.05	0.07	0.66
25. I receive support and help from my co-workers.	-0.02	0.00	0.06	0.03	0.82	0.72
26. I am treated with respect by my co-workers.	0.02	0.01	-0.02	0.01	1.01	0.78
27. There is a good relationship between me and my co-workers.	-0.01	0.03	-0.03	0.01	0.93	0.77

### Validation

CFA of the five-factor model using the final 27 items forming the iWorkHealth instrument resulted in acceptable fit (RMSEA = 0.08, TLI = 0.94, CFI = 0.95). The Cronbach’s α coefficient for the job demand, job control, employee and management engagement, supervisor support and colleague support were 0.79, 0.83, 0.86, 0.92, and 0.90, respectively. We concluded that the internal consistency of the iWorkHealth was good. The convergent validity of the instrument with other existing scales was also examined. Prior to the convergent validity analyses, the five subscale scores were obtained by summing the chosen response from relevant items within each factor. Higher scores indicate greater psychosocial risks experienced by employees. High job demand score was found to be significantly and positively associated with high burnout and depression as measured by the CBI and the PHQ-2 scores, while high job control, employee and management engagement, supervisor support and coworker support were significantly and negatively associated with burnout and depression. We also found that the convergent validity was acceptable across subgroups by age, gender, ethnicity and educational status (Table 3).

**Table 3:** Convergent validity of iWorkHealth subscales with external scales.

		Job control	Job Demand	Employee and management engagement	Supervisor support	Coworker support
All	Job Control	1	-0.23**	0.50**	0.51**	0.42**
	Job Demand	-0.23**	1	-0.36**	-0.27**	-0.17**
	Employee and management engagement	0.50**	-0.36**	1	0.53**	0.38**
	Supervisor Support	0.51**	-0.27**	0.53**	1	0.40**
	Coworker Support	0.42**	-0.17**	0.38**	0.40**	1
						
	Copenhagen Burnout Inventory	-0.44**	0.69**	-0.49**	-0.38**	-0.25**
	Personal Health Questionnaire	-0.36**	0.32**	-0.27**	-0.24**	-0.24**
	Copenhagen Burnout Inventory					
Subgroups						
Age group	Young	-0.44**	0.68**	-0.47**	-0.39**	-0.20**
	Middle	-0.41**	0.69**	-0.48**	-0.39**	-0.34**
	Older	-0.27**	0.72**	-0.44**	-0.37**	-0.36**
Gender	Female	-0.40**	0.69**	-0.48**	-0.37**	-0.22
	Male	-0.51**	0.69**	-0.49**	-0.41**	-0.31**
Ethnicity	Chinese	-0.45**	0.71**	-0.48**	-0.35**	-0.21**
	Malay	-0.35**	0.65**	-0.48**	-0.44**	-0.34**
	Indian	-0.41**	0.63**	-0.47**	-0.38**	-0.26**
	Others	-0.42**	0.68**	-0.53**	-0.43**	-0.38**
Education	Secondary and below	-0.31**	0.65**	-0.52**	-0.43**	-0.27**
	Vocational/ITE	-0.35**	0.60**	-0.41**	-0.40**	-0.37**
	Diploma	-0.43**	0.71**	-0.43**	-0.39**	-0.30**
	University and above	-0.48**	0.70**	-0.49**	-0.36**	-0.24**
	Personal Health Questionnaire					
Age group	Young	-0.35**	0.29**	-0.23**	-0.25**	-0.20**
	Middle	-0.33**	0.34**	-0.28**	-0.26**	-0.31**
	Older	-0.35**	0.15	-0.18**	-0.18**	-0.28**
Gender	Female	-0.33**	0.32**	-0.26**	-0.25**	-0.23**
	Male	-0.43**	0.29**	-0.27**	-0.24**	-0.25**
Ethnicity	Chinese	-0.39**	0.32**	-0.28**	-0.25**	-0.23**
	Malay	-0.34**	0.31**	-0.27**	-0.28**	-0.29**
	Indian	-0.25**	0.22**	-0.13*	-0.13*	-0.14**
	Others	-0.33**	0.34**	-0.27**	-0.35**	-0.33**
Education	Secondary and below	-0.28**	0.31**	-0.30**	-0.27**	-0.31**
	Vocational/ITE	-0.30**	0.21**	-0.25**	-0.27**	-0.24**
	Diploma	-0.39**	0.37**	-0.26	-0.24	-0.22
	University and above	-0.39**	0.31**	-0.26**	-0.24**	-0.21**

* = P value < 0.05;

** = P value <0.001.

### Association between socio-demographic factors and iWorkHealth subscales

After controlling for all sociodemographic factors in multivariate regression models, we identified 31 significant associations between sociodemographic factors and iWorkHealth subscales (Table 4: Model 1). Older age group was significantly associated with lower job demand and higher job control, co-worker support and employee and management engagement while the middle age group was significantly associated with higher job control and employee and management engagement than the younger age group. Females were significantly associated with lower job control, supervisor and co-workers support and employee and management engagement than males. Indians were significantly associated with lower job demand and higher job control, supervisor support and employee and management engagement than Chinese. Those with secondary education and below and vocational/ITE qualifications were significantly associated with lower job demand and co-worker support while those with diploma were significantly associated with higher employee and management engagement than those with a university degree. Those who were married were significantly associated with higher job control and employee and management engagement than those who were single. Those working in banking sector were significantly associated with lower job demand and higher supervisor and co-worker support while those working in legal sector were significantly associated with lower job control and higher employee and management engagement than those in the healthcare sector.

**Table 4:** Relationship between socio-demographic factors and iWorkHealth subscales before (model 1) and after controlling for significant DIF (model 2).

		Model 1			Model 2		
Sociodemographic correlates		B	SE	p value	B	SE	p value
		Job demand					
Age group	Middle	-0.03	0.02	0.209	0.01	0.03	0.749
(Reference: Young)	Older	-0.11	0.02	<0.001	-0.10	0.02	<0.001
Gender	Female vs. Male	-0.01	0.02	0.708	-0.01	0.02	0.706
Ethnicity	Malay	0.04	0.03	0.141	0.04	0.03	0.141
(Reference: Chinese)	Indian	-0.08	0.02	<0.001	-0.08	0.02	<0.001
	Others	0.03	0.02	0.241	0.05	0.02	0.043
Education	Vocational/ITE	-0.05	0.03	0.041	-0.05	0.03	0.041
(Reference: University)	Secondary and below	-0.06	0.03	0.014	-0.06	0.03	0.014
	Diploma	-0.04	0.02	0.133	-0.02	0.02	0.391
Marital status	Married	0.00	0.02	0.865	0.02	0.02	0.337
	Separated/Divorced	0.02	0.02	0.485	0.02	0.02	0.484
Sector	Legal	0.01	0.02	0.835	0.01	0.02	0.836
(Reference: Healthcare)	Banking	-0.09	0.02	<0.001	-0.12	0.02	<0.001
		Job control					
Age group	Middle	0.11	0.02	<0.001	0.13	0.02	<0.001
(Reference: Young)	Older	0.13	0.02	<0.001	0.14	0.03	<0.001
Gender	Female vs. Male	-0.04	0.02	0.038	-0.04	0.02	0.038
Ethnicity	Malay	0.06	0.02	0.010	0.06	0.02	0.010
(Reference: Chinese)	Indian	0.12	0.02	<0.001	0.12	0.02	<0.001
	Others	0.08	0.02	<0.001	0.08	0.02	<0.001
Education	Vocational/ITE	-0.04	0.02	0.134	-0.04	0.02	0.135
(Reference: University)	Secondary and below	-0.03	0.03	0.183	-0.06	0.03	0.027
	Diploma	-0.02	0.02	0.379	-0.04	0.02	0.084
Marital status	Married	0.09	0.02	<0.001	0.09	0.02	<0.001
	Separated/Divorced	0.03	0.02	0.141	0.03	0.02	0.141
Sector	Legal	-0.05	0.02	0.036	-0.02	0.03	0.468
(Reference: Healthcare)	Banking	0.02	0.02	0.402	0.00	0.02	0.961
		Supervisor support					
Age group	Middle	-0.03	0.02	0.273	-0.03	0.02	0.273
(Reference: Young)	Older	0.04	0.02	0.072	0.04	0.02	0.072
Gender	Female vs. Male	-0.06	0.02	0.007	-0.06	0.02	0.007
Ethnicity	Malay	-0.02	0.03	0.433	-0.02	0.03	0.433
(Reference: Chinese)	Indian	0.08	0.02	<0.001	0.08	0.02	<0.001
	Others	0.04	0.02	0.045	0.04	0.02	0.045
Education	Vocational/ITE	0.00	0.02	0.876	0.00	0.02	0.876
(Reference: University)	Secondary and below	-0.03	0.03	0.330	-0.03	0.03	0.330
	Diploma	-0.01	0.02	0.732	-0.01	0.02	0.732
Marital status	Married	0.03	0.02	0.224	0.03	0.02	0.224
	Separated/Divorced	-0.02	0.02	0.351	-0.02	0.02	0.351
Sector	Legal	0.02	0.02	0.358	0.02	0.02	0.358
(Reference: Healthcare)	Banking	0.05	0.02	0.011	0.05	0.02	0.011
		Co-worker support					
Age group	Middle	-0.03	0.03	0.287	-0.03	0.03	0.287
(Reference: Young)	Older	0.07	0.03	0.004	0.07	0.03	0.004
Gender	Female vs. Male	-0.05	0.02	0.048	-0.05	0.02	0.048
Ethnicity	Malay	-0.01	0.03	0.786	-0.01	0.03	0.786
(Reference: Chinese)	Indian	0.03	0.02	0.229	0.03	0.02	0.229
	Others	0.02	0.02	0.476	0.02	0.02	0.476
Education	Vocational/ITE	-0.07	0.03	0.010	-0.07	0.03	0.010
(Reference: University)	Secondary and below	-0.13	0.03	<0.001	-0.13	0.03	<0.001
	Diploma	-0.05	0.02	0.053	-0.05	0.02	0.053
Marital status	Married	0.03	0.02	0.268	0.03	0.02	0.268
	Separated/Divorced	-0.01	0.02	0.825	-0.01	0.02	0.825
Sector	Legal	0.00	0.02	0.946	0.00	0.02	0.946
(Reference: Healthcare)	Banking	0.05	0.02	0.045	0.05	0.02	0.045
		Employee and management engagement					
Age group	Middle	0.10	0.02	<0.001	0.10	0.02	<0.001
(Reference: Young)	Older	0.17	0.02	<0.001	0.17	0.02	<0.001
Gender	Female vs. Male	-0.06	0.02	0.003	-0.06	0.02	0.003
Ethnicity	Malay	0.00	0.03	0.939	0.00	0.03	0.939
(Reference: Chinese)	Indian	0.10	0.02	<0.001	0.10	0.02	<0.001
	Others	0.10	0.02	<0.001	0.10	0.02	<0.001
Education	Vocational/ITE	-0.01	0.02	0.805	-0.01	0.02	0.805
(Reference: University)	Secondary and below	0.02	0.03	0.530	0.02	0.03	0.530
	Diploma	0.05	0.02	0.023	0.05	0.02	0.023
Marital status	Married	0.05	0.02	0.020	0.05	0.02	0.020
	Separated/Divorced	-0.01	0.02	0.829	-0.01	0.02	0.829
Sector	Legal	0.06	0.02	0.010	0.06	0.02	0.010
(Reference: Healthcare)	Banking	-0.03	0.02	0.144	-0.03	0.02	0.144

Model 1 beta coefficients were derived from direct effects of sociodemographic factors on iWorkHealth subscales in MIMIC model

Model 2 beta coefficients were derived from direct effects of sociodemographic factors on iWorkHealth subscales in MIMIC model after adjusting for significant DIF items

### Differential item functioning

We found that 10 items from the job demand and job control dimensions had significant DIF in relation to age, ethnicity, education level and sector (Table 5). For example, item 10 “my work in meaningful” and item 11 “my work is important” exhibited DIF in relation to sector i.e., those in the legal sector tend to have lower probability of endorsement of item 10 and item 11 than those in the healthcare sector.

**Table 5:** Significant direct relationship between items and covariates in the MIMIC model.

Subscales	Items	Sociodemographic factors	B	SE	p value
Job demand	I have so much work to do that I am unable to do a good job	Ethnicity: Others vs. Chinese	-0.07	0.019	<0.001
	3. I still feel tired from the previous work day / shift even as I start the next one.	Age group: Middle vs. Young	-0.12	0.018	<0.001
		Age group: Older vs. Young	-0.07	0.019	<0.001
		Marital status: Married vs. Single	-0.09	0.019	<0.001
	4. My work is emotionally demanding.	Age group: Older vs. Young	0.06	0.022	0.006
		Education: Diploma vs. University	-0.08	0.020	<0.001
	5. In my work, I experience contradictory demands.	Sector: Banking vs. Healthcare	0.10	0.020	<0.001
Job control	6. I know exactly what is expected of me at work.	Sector: Banking vs. Healthcare	0.11	0.017	<0.001
		Education: Secondary and below vs. University	0.15	0.023	<0.001
		Education: Diploma vs. University	0.14	0.022	<0.001
	7.I can use my skills and expertise in my job.	Sector: Legal vs. Healthcare	0.05	0.020	0.011
		Sector: Banking vs. Healthcare	0.07	0.018	<0.001
	8.I have enough information to get my job done.	Sector: Legal vs. Healthcare	0.12	0.022	<0.001
	9.I receive enough help and equipment to get my job done.	Age group: Middle vs. Young	-0.14	0.022	<0.001
		Age group: Older vs. Young	-0.08	0.022	<0.001
		Sector: Legal vs. Healthcare	0.10	0.022	<0.001
	10.My work is meaningful.	Sector: Legal vs. Healthcare	-0.22	0.018	<0.001
		Sector: Banking vs. Healthcare	-0.05	0.015	<0.001
	11.My work is important.	Sector: Legal vs. Healthcare	-0.12	0.019	<0.001

## Discussion

This paper describes the development of a psychometrically sound, robust and easily administrable iWorkHealth instrument which considered all the culturally relevant domains of psychosocial risks in a multi-ethnic Asian working population in Singapore. The iWorkHealth identified five key dimensions of psychosocial risk factors in Singapore. Results from the EFA and MSA showed that they were associated with dimensions of job demand, job control, employee and management engagement, supervisor support and colleague support. Some of these dimensions were quite similar with those reported in the literature [6, 7, 23], such as job demand, job control, supervisor support and co-worker support while others such as employee and management engagement emerged as salient in the local population. Our results showed that the job demand dimension was associated with 5 items measuring high workload—so much work to do, feeling tired after work/shift-work, work that is emotionally demanding, and experience contradictory demands. The job control dimension was clearly associated with 7 items measuring whether the job met their expectation, skills and expertise, whether they had enough information to get the job done, whether they perceived their job as meaningful and important as well as feeling that enough help and equipment was available, and whether they felt motivated and involved in their work. Apart from that, the analyses clearly showed that the instrument was able to differentiate items that measured support from co-workers (3 items) and supervisor (5 items) and demonstrated that the two dimensions are distinct. The employee and management engagement dimension was clearly associated with 7 items measuring satisfaction with the pay and benefits received and opportunities for promotion, whether rewards were given fairly, whether company treated employees well, whether employees received the respect and prestige they deserved, whether company considered employee welfare much more important than operations/sales and profit and whether company managed change well.

The internal reliability of the instrument was supported by Cronbach’s alpha results. The value of the Cronbach’s alpha for each scale was above the cutoff of 0.70 [24]. The validity of the instrument was also strongly supported by convergent validity of the subscales with other existing scales which measured burnout and depression. In this analysis we found that high job demand was strongly and directly associated with burnout and depression. Meanwhile, high job control, supervisor support, co-worker support, employee and management engagement were inversely associated with the burnout and depression scores. According to the Job Demand Control model, high job demand and low job control needed to exist simultaneously in order to produce high job strain [23] that could predict mental illnesses [6]. In our study, high job demand and low job control were independently associated with the burnout and depression scores. Hence it strongly supported the Job Demand Control model. We incorporated analyses based on key sociodemographic factors and identified 31 significant associations between sociodemographic factors and iWorkHealth subscales. These findings suggest that the instrument seems to have good properties to differentiate the multi-faceted nature of workplace in our sample across age, gender, ethnicity, marital status, education level and sector. However, we identified 10 items from job demand and job control dimensions that exhibited significant DIF in relation to age, ethnicity, education level and sector. Hence, we implemented MIMIC modelling to address DIF and reduce its impact on group comparisons. After inclusion of significant DIF in the multivariate regression models within the MIMIC framework (Table 4: Model 2), we found that only two associations became non-significant. All the other 29 associations remained significant which suggests that the impact of DIF was minimal.

While the current instrument demonstrated superior reliability and validity in Singapore’s working population, some limitations of this study should be addressed. The study was limited to English speaking adults, those who were working in healthcare, banking and finance, and legal sectors. The 5 firms participated in the study voluntarily. Therefore, the findings may not representative of all firms in Singapore. Moreover, other dimensions of psychosocial risk factors in the wider population such as those from construction or teaching sectors may have fallen outside the scope of this study. Our study had used a cross-sectional design, thus preventing us from concluding any causal relationship between the psychosocial risk factors at the workplace and self-perceived burnout and depression. However, findings from other studies using longitudinal research design, have suggested possible relationship between psychosocial risk factors at workplace and self-perceived depression [25]. Another limitation of this study was that all of the study data were self-reported which may have introduced bias. However, self-report is often the only feasible strategy to gather information concerning workers' psychosocial risk factors [26].

Overall, the findings provided evidence that the iWorkHealth instrument, which comprises 27 items in five domains of workplace psychosocial risk factors, was reliable and valid for providing insights on the prevalence of psychosocial risk factors and their relationships with employee well-being in the workplace. Subsequently it can be used to provide a normative base to identify successful interventions, contributing to the development of best practice, standards and guidelines for the management of psychosocial risk factors in the workplace in Singapore.

## Supporting information

S1 File iWorkHealth instrument. (DOCX)
