# Supplementary material for: iWorkHealth: An instrument to identify workplace psychosocial risk factors for a multi-ethnic Asian working population
Source: PLoS One. 2019 Aug 7;14(8):e0220566. doi: 10.1371/journal.pone.0220566 (PMC6685634; doi:10.1371/journal.pone.0220566)
Supplement: S1 File — (DOCX) [file pone.0220566.s001.docx]

Instructions

1. Answer all questions.
2. Give your first and natural response, do not spend too much time on a question.
3. For each question, please CIRCLE one option that you think most closely applies to you. Try not to choose “Not Applicable”, unless it is really the case.

| No. | **How are your current job, colleagues, supervisors and workplace?** | **Not applicable** | **Strongly Disagree** | **Disagree** | **Neither Agree Nor Disagree** | **Agree** | **Strongly Agree** |
| --- | --- | --- | --- | --- | --- | --- | --- |
| 1 | I feel that my workload is too heavy. | 0 | 1 | 2 | 3 | 4 | 5 |
| 2 | I have so much work to do that I am unable to do a good job | 0 | 1 | 2 | 3 | 4 | 5 |
| 3 | I still feel tired from the previous work day / shift even as I start the next one. | 0 | 1 | 2 | 3 | 4 | 5 |
| 4 | My work is emotionally demanding. | 0 | 1 | 2 | 3 | 4 | 5 |
| 5 | In my work, I experience contradictory demands. | 0 | 1 | 2 | 3 | 4 | 5 |
| 6 | I know exactly what is expected of me at work. | 0 | 1 | 2 | 3 | 4 | 5 |
| 7 | I can use my skills and expertise in my job. | 0 | 1 | 2 | 3 | 4 | 5 |
| 8 | I have enough information to get my job done. | 0 | 1 | 2 | 3 | 4 | 5 |
| 9 | I receive enough help and equipment to get my job done. | 0 | 1 | 2 | 3 | 4 | 5 |
| 10 | My work is meaningful. | 0 | 1 | 2 | 3 | 4 | 5 |
| 11 | My work is important. | 0 | 1 | 2 | 3 | 4 | 5 |
| 12 | I feel motivated and involved in my work. | 0 | 1 | 2 | 3 | 4 | 5 |
| 13 | I receive the respect and prestige I deserve at work. | 0 | 1 | 2 | 3 | 4 | 5 |
| 14 | I am satisfied with the amount of pay and benefits I receive. | 0 | 1 | 2 | 3 | 4 | 5 |
| 15 | I find the opportunities for promotion within the company are good. | 0 | 1 | 2 | 3 | 4 | 5 |
| 16 | I feel that rewards for my effort are given in a fair way. | 0 | 1 | 2 | 3 | 4 | 5 |
| 17 | I feel this company treats its employees well. | 0 | 1 | 2 | 3 | 4 | 5 |
| 18 | I think this company considers employee welfare much  more important than operations / sales and profits. | 0 | 1 | 2 | 3 | 4 | 5 |
| 19 | My company manages changes in policies / structures / processes well. | 0 | 1 | 2 | 3 | 4 | 5 |
| 20 | I receive support and guidance from my immediate supervisor. | 0 | 1 | 2 | 3 | 4 | 5 |
| 21 | My immediate supervisor is concerned about the welfare of his or her staff. | 0 | 1 | 2 | 3 | 4 | 5 |
| 22 | My immediate supervisor is successful in getting people to work together. | 0 | 1 | 2 | 3 | 4 | 5 |
| 23 | I am treated with respect by my immediate supervisor. | 0 | 1 | 2 | 3 | 4 | 5 |
| 24 | My immediate supervisor talks with me about how well I carry out my work. | 0 | 1 | 2 | 3 | 4 | 5 |
| 25 | I receive support and help from my co-workers. | 0 | 1 | 2 | 3 | 4 | 5 |
| 26 | I am treated with respect by my co-workers. | 0 | 1 | 2 | 3 | 4 | 5 |
| 27 | There is a good relationship between me and my co-workers. | 0 | 1 | 2 | 3 | 4 | 5 |
